# Prescribing patterns and compliance with World Health Organization recommendations for the management of severe malaria: a modified cohort event monitoring study in public health facilities in Ghana and Uganda

**DOI:** 10.1186/s12936-019-2670-9

**Published:** 2019-02-08

**Authors:** H. Hilda Ampadu, Kwaku Poku Asante, Samuel Bosomprah, Samantha Akakpo, Pierre Hugo, Helga Gardarsdottir, Hubert G. M. Leufkens, Dan Kajungu, Alexander N. O. Dodoo

**Affiliations:** 1 The African Collaborating Centre for Pharmacovigilance & Surveillance, Accra, Ghana; 2 Division of Pharmacoepidemiology and Clinical Pharmacology, Utrecht Institute for Pharmaceutical Sciences, Faculty of Science, Utrecht University, Utrecht, The Netherlands; 3 Kintampo Health Research Centre, Ghana Health Service, Kintampo, Ghana; 4 Department of Biostatistics, School of Public Health, University of Ghana, Accra, Ghana; 5 Medicines for Malaria Venture, Geneva, Switzerland; 6 Department of Clinical Pharmacy, Division Laboratory and Pharmacy, University Medical Centre Utrecht, Utrecht, The Netherlands; 7 Makerere University Centre for Health and Population Research (MUCHAP), Iganga/Mayuge Health and Demographic Surveillance Site (IMHDSS), Kampala, Uganda

**Keywords:** Prescription, Malaria, Injectable artesunate, Injectable artemether, Injectable quinine

## Abstract

**Background:**

Injectable artesunate (AS) is the World Health Organization (WHO) recommended medication for the treatment of severe malaria followed with an oral artemisinin-based combination therapy (ACT). There are few studies indicating how physicians prescribe injectable AS, injectable quinine (Q) or injectable artemether (AR) and ACT for severe malaria. This study was undertaken to evaluate prescription compliance to the WHO recommendation in 8 public health facilities in Ghana and Uganda. This was a modified cohort event monitoring study involving patients who were administered with injectable anti-malarial for treatment of presumed or confirmed severe malaria. Patients prescribed at least one dose of injectable artesunate, artemether or quinine qualified to enrol in the study. Patients were recruited at inpatient facilities and followed up in the hospital, by phone or at home. Following WHO recommendations, patients are to be prescribed 3 doses of injectable AS, Q or AR for at least 24 h followed with oral ACT. Compliance rate was estimated as the number of patient prescriptions that met the WHO recommendation for treatment of severe malaria divided by the total number of patients who completed the study by end of follow up. Log-binomial regression model was used to identify predictors for compliance. Based on the literature and limitations of available data from the patients’ record, the diagnosis results, age, gender, weight, and country were considered as potential predictors of prescriber adherence to the WHO recommendations.

**Results:**

A total of 1191 patients completed the study, of which 93% were prescribed injectable AS, 3.1% (injectable AR or Q) with 32.5% prescribed follow-on oral ACT and 26% on concomitant antibiotics. 391 (32.8%) were in Ghana and 800 (67.2%) in Uganda. There were 582 (48.9%) women. The median age was 3.9 years (IQR = 2, 9) and median weight was 13 kg (IQR = 10, 20). Of the 1191 patients, 329 of the prescriptions complied with the WHO recommendation (compliance rate = 27.6%; 95% CI = [25.2, 30.2]). Diagnostic results (Adjusted prevalence ratio (aPR) = 4.56; 95% = [3.42, 6.08]; p < 0.0001) and weight (20 + kg vs < 10 kg: aPR = 0.65; 95% = [0.44, 0.96]; p = 0.015) were identified as factors independently associated with compliance.

**Conclusion:**

Injectable AS is the most commonly prescribed medicine in the management of severe malaria in Ghana and Uganda. However, adherence to the WHO recommendation of at least 3 doses of injectable anti-malarial in 24 h followed by a full course of ACT is low, at less than 30%.

## Background

Over the past 10 years, there has been a huge decline in malaria cases as well as deaths from malaria. According to the World Malaria Report, there has been a 22% reduction in malaria cases between 2010 and 2017 [1], accompanied by a huge decline in malaria deaths ranging from 10% in the Eastern Mediterranean through 40% in Africa to 54% in South-East Asia. In spite of these huge reductions, 435,000 deaths due to malaria were reported in 2017, compared with 451,000 estimated deaths in 2016, and 607,000 in 2010. This shows that modest progress has reduced compared to the previous year. The vast majority (more than 90%) of these estimated malaria deaths occurred in sub-Saharan Africa. Global efforts set out in the Global Technical Strategy (GTS) for Malaria [2] as well as the Roll Back Malaria advocacy plan “Action and investment to defeat malaria” [3] aim to reduce malaria morbidity and mortality by at least 90% by 2030 compared to a 2015 baseline to meet the Sustainable Development Goals, [4]. Most malaria deaths are attributed to infections by *Plasmodium falciparum.* Poorly managed *P. falciparum* infections can lead to severe malaria which is associated with extremely high case fatality rates averaging 16–20% though extreme ranges as low as 2% and high as 100% have also been reported [5] these extremes being inconsistent with the overall literature.

The World Health Organization (WHO) recommendation for severe malaria is the appropriate use of injectable artesunate [6]. This recommendation is backed by a strong body of evidence, including the landmark SEAQUAMAT [7] and AQUAMAT [8] studies, as well as a Cochrane review [9]. When injectable artesunate (AS) is not available, parenteral artemether (AR) or quinine (Q) are recommended. When used correctly, injectable AS, AR or Q are highly efficacious. The WHO also recommends that the parenteral medication is followed by a full course of oral artemisinin-based combination therapy (ACT) to complete the treatment for severe malaria. However, there is little evidence of adherence to these recommendations in routine practice.

In a study in Swaziland covering the period 2011–2015, where patients were diagnosed either by rapid diagnostic tests (RDTs), microscopy or both [10], less than half of those with severe malaria received injectable anti-malarial and an oral course of ACT. Fourteen percent of the 1981 patients who had severe malaria were treated with artemether-lumefantrine alone (11%), an ACT meant for treating uncomplicated malaria and not severe malaria. The rest were treated either with quinine alone (44%) or a combination of quinine and artemether-lumefantrine (45%), i.e. an injectable anti-malarial and an oral ACT. A cross-sectional survey of inpatient malaria investigation involving 13,014 children admitted to 5 hospitals in Western Kenya between 2014 and 2016 revealed high rates of presumptive treatment and possible over-use of injectable anti-malarials [11]. Another study in rural Western Kenya in 2013 showed poor knowledge and incorrect prescribing in the treatment of malaria in pregnancy [12].

In view of the high case fatality rate associated with severe malaria, it is important to understand the routine management of patients with severe malaria and to assess whether prescribing patterns are in line with recommended guidelines. The focus of this manuscript is on prescribers’ compliance to the WHO recommendations for treating patients with severe malaria especially considering the paucity of publications on prescription adherence to recommended treatment for severe malaria.

This study sought to describe medicine prescription in public health facilities in Ghana and Uganda regarding WHO guidance on the treatment of severe malaria to find out the level of compliance to the recommendations. It also examined factors independently associated with compliance to the WHO recommendation. The findings provide insight into what pertains in real-world settings in relation to WHO guidance on treatment of severe malaria in particular in the use of the WHO recommended approach of prescribing injectable anti-malarials (AS, AR or Q) followed by a full course of oral ACT for the treatment of severe malaria.

## Methods

### Study design and participants

This was a modified cohort event monitoring study involving patients who were prescribed injectable anti-malarial for treatment of presumed severe malaria in 8 sites (4 each in Ghana and Uganda) between May and December 2016. The full details of the methods have been previously published [13]. In short, eligible patients being treated for presumed severe malaria were recruited following signed informed consent. Patients were eligible for inclusion if they had severe/complicated malaria (*Plasmodium* of any species) presumed or diagnosed as per national policies and health facility practice/protocol [6]; if they were able and willing to participate in the study; and if they agreed to the schedule for follow-up contact or home visits. Patients were excluded if they had serious concurrent illness. All eligible patients gave informed consent. For children, informed consent was obtained from parents or a caregiver/guardian.

Data on medicine prescription were extracted from patients’ records kept in paper “folders” in each health facility together with physician clerking notes, nurses’ medicines administration records and pharmacy medicine supply details. Since the criteria for enrolment in this study was the prescription of injectable AS, AR or Q, all patients in the study had at least one dose of these medicines prescribed in addition to any concomitant medications prescribed. Data was extracted from the patient medical files on method of diagnosis, diagnosis or test results, date of treatment, age, gender, dose and frequency of anti-malarial as well as the prescribed route of administration. Data on laboratory results was important as the WHO recommendations require 12-hourly microscopy to monitor response to treatment.

### Sample size calculation

The sample size calculation was based on the primary outcome of compliance to WHO recommendation for treatment of severe malaria. The main analysis estimated the compliance rate and its 95% confidence. Therefore, the sample size was estimated using precision approach for one proportion in PASS 16 (NCSS, LLC, Kaysville, Utah, USA). Although previous studies reported approximately 90–95% prescription of 3 doses of AS, no study reported appropriate prescription in terms of 3 doses of AS followed by ACT. However, anecdotal evidence from routine hospital data showed that this was low. Therefore, a conservative compliance rate of 27% was assumed. Based on this assumption, a sample size of 1212 produces a two-sided 95% confidence interval with a margin of error equal to ± 2.5% (equivalent to a 2-sided confidence interval width equal to 0.050) when the sample proportion is 0.27.

### Data management

Data on patient demography, the method used to test for malaria parasitaemia as part of the diagnosis of severe malaria as well as the overall management of the severe malaria episode itself were extracted from the patient records contained in paper “patient folders” in each facility. All these data were then transcribed onto the study Case Report Forms (CRFs). There were individual CRFs for collecting Demography, Medical History, Study and Concomitant Medication data, Laboratory data, the various Follow-Up information as well as individual CRFs for collecting Adverse Drug Reactions and Serious Adverse Drug Reactions data. All these CRFs formed what was called a CRF book per patient. Upon completion of a patient CRF book, the Site Coordinator as well as the Principal Investigator signed off on the book indicating that the book is complete. Data were entered, managed and stored in a specially created version of MedSpina™, an in-house electronic health records system that allows clinicians and other health workers to collect patients’ data, including laboratory results, to facilitate patient care. The Data Manager performed first pass quality control on all data points per patient CRF book by making sure data on the CRF matched the data in the database. All discrepant data points were queried for clarification/correction. Upon completion of the data management process, all data points for all patients were extracted for statistical analysis.

### Outcome

The primary outcome was compliance to the WHO recommended treatment guidelines for severe malaria [6] defined as the fraction of patient prescription that met WHO recommendation on treatment of severe malaria. The WHO guidelines specify that “anti-malarial drugs should be given parenterally for a minimum of 24 h and replaced by oral medication as soon as it can be tolerated”. For injectable AS, AR or Q, the WHO guidance means that patients diagnosed with severe malaria should be treated with at least 3 doses of injectable anti-malarial for at least 24 h and should complete treatment with 3 days of oral ACT.

### Covariates and concomitant medication

Based on the literature and limitations of available data from the patients’ record, the diagnosis results, age, gender, weight, and country were considered as potential predictors of prescription. Use of concomitant medications was defined as any other medicines taken in addition to the WHO recommended anti-malarials. Thus, medicines being taken by patients during the course of the study such as antibiotics, haematinics, and analgesics among others was considered as covariates.

### Statistical analysis

The prescribed medicines were re-coded into seven categories namely: injectable AS, AR or Q, ACT, antibiotics, analgesic or antipyretic, haematinic or vitamin, and others. The primary outcome was calculated as the number of patient prescriptions that met the WHO recommendation for treatment of severe malaria divided by the total number of patients who completed the study by end of follow up.

The log binomial regression model was used to identify factors independently associated with compliance to the WHO recommendation. To construct a parsimonious model using all the potential predictors, a model for each potential predictor was first fitted. In this model, each variable were candidates for inclusion in the full model if the p-value for association with prescription was 0.2 or less when considered individually. Variables were then removed from the model if the p-value for the likelihood ratio test was more than 0.2, provided removal did not change coefficients of variables in the model by more than 10%. Standard errors were adjusted for clustering of patients within health facilities. Missingness in the potential predictors were assumed as having occurred at random and not related to the outcome and, therefore, were not imputed. In a secondary analysis, the proportion of patients who were diagnosed with different malaria diagnostic methods were estimated. All analyses were performed using Stata 15 MP (StataCorp, College Station, Texas, USA).

## Results

### Characteristics of patients

**Fig. 1:**
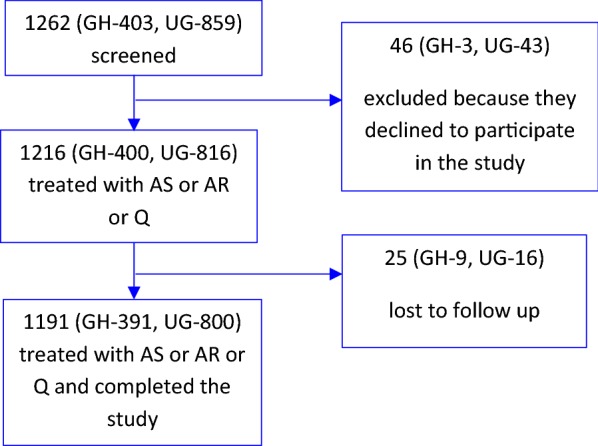
Patient flow: *GH* Ghana, *UG* Uganda

A total of 1262 patients were screened but 46 declined to participate giving 1216 patients treated with AS and or other anti-malarials and 25 having been lost to follow up leaving 1191 patients who were included in the analysis (Fig. 1). Of the 1191 remaining patients treated with injectable AS, AR, or Q and completed the study, 391 (32.8%) were in Ghana and 800 (67.2%) were in Uganda, there were 582 (48.9%) females; the median age was 3.9 years (IQR = 2, 9) and median weight was 13 kg (IQR = 10, 20) (Table 1).

**Table 1:** Proportion of patient prescription that complied with WHO recommendation by background characteristics

Characteristics	Number of patients (% of total)	Prescription of inj. AS in complied doses; n (%)			Co-prescription of ACT	# (%) of prescriptions that complied with WHO recommendation*
		< 3 doses	3 doses	> 3 doses	n (%)	n (%); [95% CI]
Sex						
Female	582 (48.9)	41 (7.0)	492 (84.5)	49 (8.4)	178 (30.6)	153 (26.3); [22.9, 30.0]
Male	609 (51.1)	43 (7.1)	526 (86.4)	40 (6.6)	196 (32.2)	176 (28.9); [25.4, 32.6]
Age (years)						
Median (IQR)	3.9 (2, 9)					
5+	476 (40.0)	33 (6.9)	386 (81.9)	57 (12.0)	119 (25.0)	101 (21.2); [17.8, 25.1]
Under 5	712 (59.7)	50 (7.0)	630 (88.5)	32 (4.5)	254 (35.7)	227 (31.9); [28.6, 35.4]
Missing	3 (0.3)	1 (33.3)	2 (66.7)	0 (0)	1 (33.3)	
Weight (kg)						
Median (IQR)	13 (10, 20)					
< 10	276 (23.2)	19 (6.9)	246 (89.1)	11 (4.0)	89 (32.3)	85 (30.8); [25.6, 36.5]
10–19	510 (42.8)	35 (6.9)	450 (88.2)	25 (4.9)	191 (37.5)	163 (32.0); [28.0, 36.1]
20–29	111 (9.3)	8 (7.2)	96 (86.5)	7 (6.3)	32 (28.8)	29 (26.1); [18.8, 35.1]
30+	200 (16.8)	14 (7.0)	149 (74.5)	37 (18.5)	23 (11.5)	16 (8.0); [4.9, 12.7]
Missing	94 (7.9)	8 (8.5)	77 (81.9)	9 (9.6)	39 (41.5)	
Country						
Ghana	391 (32.8)	31 (7.9)	355 (90.8)	5 (1.3)	336 (85.9)	301 (77.0); [72.3, 80.9]
Uganda	800 (67.2)	53 (6.6)	663 (82.9)	84 (10.5)	38 (4.8)	28 (3.5); [2.4, 5.0]
Total	1191 (100)	84 (7.1)	1018 (85.5)	89 (7.5)	374 (31.4)	329 (27.6); [25.2, 30.2]

* Compliance was defined as prescription of 3 doses of inj. AS within 24 h and treatment with ACT

### Medicine prescription

**Fig. 2:**
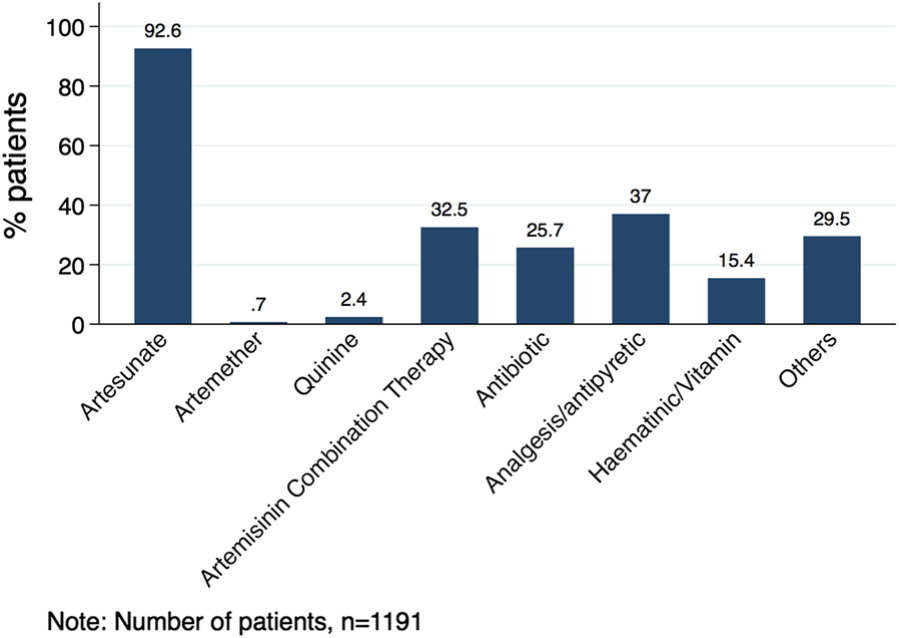
Types of medicines prescribed to patients with severe malaria

Ninety-three percent (93%) of the patients were prescribed injectable artesunate, 3.1% (injectable artemether or quinine), and 32.5% oral ACT (Fig. 2). About a third (26%) of the patients were prescribed antibiotics (Fig. 2). Of the 1191 patients treated with AS, AR, or Q, 329 (27.6%; 95% CI 25.2–30.2) of the prescriptions complied with WHO recommendation for treatment of severe malaria (Table 1). Compliance rate among children under 5 years was higher than that of children above 5 years of age (31.9% (CI 28.6–35.4) vs 21.2% (CI 17.8–25.1) (Table 1). Majority (747) of Ugandan patients were prescribed at least 3 doses of inj. AS; however, this was largely not followed with co-prescription of oral ACT (Table 1). Overall, 374 of the patients (31.4%) had injectable anti-malarial (injectable AS or AR or Q) and co-prescription of ACT (Table 1). While about one-third (254/712) of children under 5 years had injectable anti-malarial plus co-prescription of ACT, only 4.8% of patients in Uganda had injectable anti-malarial (all of them injectable AS) plus co-prescription of oral ACT (Table 1). 1018 (85.5%) of the patients had 3 doses of injectable AS (Table 1).

### Factors independently associated with compliance of prescribers with WHO recommendation

In univariable analyses, compliance to WHO recommendations for patients diagnosed as negative was about 4 times that among those diagnosed as positive whereas compliance to WHO recommendation for patients under 5 years of age was about 50% higher compared to children above 5 years (Table 2). Regarding weight, compliance to prescription among heavier patients was lower than that among lighter patients. There was no evidence that gender has any association with compliance (Table 2). In multivariable analysis where all factors have been adjusted for, the association of diagnostic results (Adjusted prevalence ratio (aPR) = 4.56; 95% = [3.42, 6.08]; p < 0.0001) and weight (20 + kg vs < 10 kg: aPR = 0.65; 95% = [0.44, 0.96]; p = 0.015) with compliance remained statistically significant at 5% level of significance (Table 2).

**Table 2:** Factors independently associated with compliance of patient prescription with WHO recommendation (n = 1094)

Factors	Crude PR [95% CI]	LR p-value	Adjusted PR [95% CI]	LR p-value
Diagnosis result				
Positive	Ref	*< 0.0001*	Ref	*< 0.0001*
Negative	4.16 [3.20, 5.41]		4.56 [3.42, 6.08]	
Sex				
Female	Ref	0.314		
Male	1.10 [0.91, 1.32]			
Age (years)				
5+	Ref	*< 0.0001*	Ref	0.205
Under 5	1.50 [1.23, 1.84]		1.16 [0.89, 1.51]	
Weight (kg)				
< 10	Ref	*< 0.0001*	Ref	*0.015*
10–19	1.03 [0.83, 1.29]		1.08 [0.88, 1.32]	
20+	0.47 [0.34, 0.65]		0.65 [0.44, 0.96]	

*PR* prevalence ratio

### Diagnosis of severe malaria

**Fig. 3:**
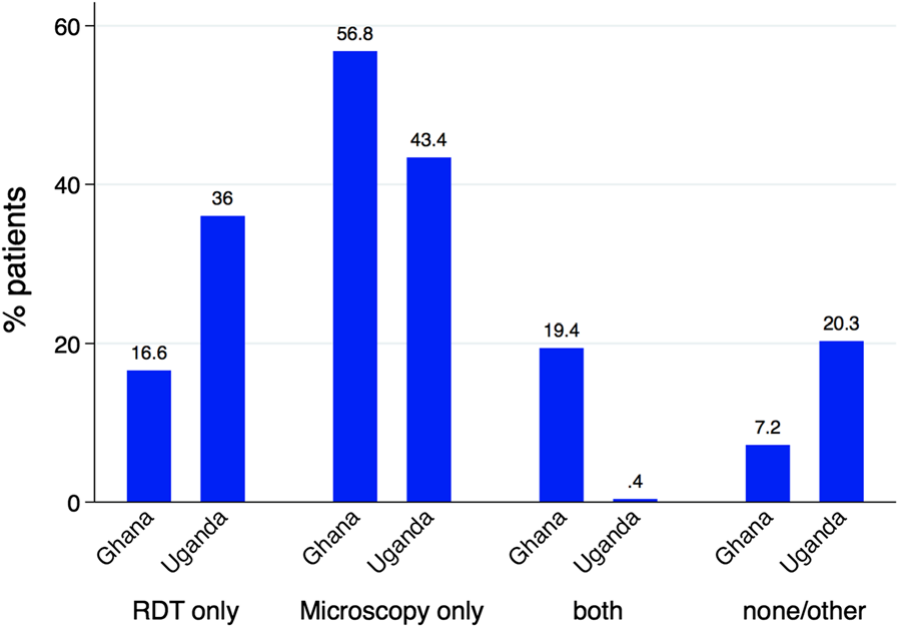
Proportion of patients diagnosed with different malaria diagnostic methods

Of the 1191 patients, 569 (47.8%) were tested for parasitaemia using microscopy only whereas 353 (29.6%) were tested using RDT only. For each setting, 56.8% (222/391) of patients in Ghana were tested using microscopy only compared to 43.4% (347/800) in Uganda whereas 16.6% (65/391) of the Ghanaian cohort were tested using RDT only compared to 36.0% (288/800) in Uganda (Fig. 3). About 19.4% of patients in Ghana were tested using both RDT and Microscopy compared to 0.4% in Uganda (Fig. 3). None of the patients had 12-h microscopy to monitor outcome.

## Discussion

This study reveals prescribers’ prescription practices for patients treated for suspected severe malaria in 8 public health facilities in Ghana and Uganda. There was a very high level of prescription of injectable AS with 93% of the 1191 patients being prescribed the product. The rest were prescribed injectable AR or Q. Whilst 85.5% of the patients were prescribed 3 doses of injectable AS, only 31.4% had prescriptions for injectable AS followed by oral ACT. This shows a rather low level of compliance to the WHO recommendations for treating severe malaria which recommends the use of an injectable anti-malarial for 24 h (which equates to at least 3 doses of injectable AS, AR or Q) followed by oral ACT. There was high differences between countries with compliance being 20 times higher in Ghana compared to Uganda where only 4.8% of patients had a prescription for an injectable anti-malarial followed by a co-prescription of oral ACT. This finding contrasts with that of Achan et al. [14] where 429 out of 823 patients who received parenteral anti-malarial also received oral medication. Even though there is a possibility that some patients may have received prescriptions for oral medications which were not captured in their in-patient folders and were also not reported to the study team for inclusion in the CRFs, it is extremely doubtful that this is frequent enough to reflect in the low follow-on ACT prescribed. The very high prescription of injectable AS, the WHO recommended treatment, across the public health facilities in the 2 countries indicates its acceptance as the gold standard for treating severe malaria. However, there is the need to improve education to ensure that the “at least 24-h parenteral treatment” is followed by a full course of oral ACT. Previous authors have shown poor practices in relation to the management of severe malaria. A 2009 severe malaria case study in Uganda concluded that management of severe malaria was poor with the correct drug at the time (quinine) being prescribed but used either mixed incorrectly or dosed sub-optimally [14]. The authors concluded that only 16.9% of the 868 patients were appropriately treated for severe malaria.

In this study, prescribers appeared to pay particular attention to children under 5 years in respect of compliance with the WHO recommendations. The findings showed a 20% increase in compliance among prescribers in relation to children under 5 years of age compared to older patients, suggesting a predilection towards more careful management of these children considered as high risk group. Although not statistically significant at 5% level in this study, compliance when prescribing for those who were negative for malaria parasites was about 15% higher than those positive for malaria parasites. Prescribing anti-malarials for patients without parasitaemia is well known, both for severe as well as uncomplicated malaria and in one study in Kenya in 2016, 69% of patients with negative malaria tests were still given parenteral treatment [11]. An earlier study in Uganda from 2011 to 2013 involving 58,095 children revealed similar practices [15]. The WHO treatment guidelines advises prescribers not to withhold anti-malarial treatment from patients whilst waiting for parasitological confirmation of malaria even though it advises prescribers to look for other potential causes of the admission. Patients were prescribed anti-malarials even when parasitological tests for malaria were negative. Prescribers tend to err on the side of caution to offer treatment even when malaria tests are negative though several studies have shown that withholding treatment in cases where malaria tests are negative is safe [16, 17]. A reason for prescribing anti-malarials for patients who tested negative for malaria may be that the attending physicians may want to treat for severe malaria in the absence of any other obvious clinical diagnosis. In patients with no malaria parasites, the treating physicians seeing no obvious cause for the clinical state rather appears to rigorously follow the WHO guidelines for treating severe malaria whilst also exploring treatment for other conditions which may be co-existing. In fact, the WHO guidelines for treating severe malaria highlights the similarities and co-existence of severe malaria, pneumonia and septicaemia and recommends simultaneous treatment for all these even before laboratory results are obtained. This may also explain the relatively high (26%) concomitant prescription of antibiotics in this study. Studies on drug utilization in uncomplicated malaria [18] have shown similar results with co-prescription of antibiotics being high among patients with febrile illness.

The main medicines co-prescribed with the anti-malarial treatments were analgesics (37%), antibiotics (26%) and haematinics (15%). Analgesics and haematinics are routinely prescribed for both uncomplicated and severe malaria to treat fever and anaemia, respectively. As explained above, the use of antibiotics may be justified by the fact that attending physicians may be treating for other conditions which may be co-existing with the severe malaria. The drug prescription pattern in this study can be compared with those of a similar study on prescribing for patients with uncomplicated malaria where 31% and 26% received antibiotics and vitamins, respectively [18]. Such prescribing may be pragmatic in the treatment of severely ill patients in settings where diagnostics may also not be readily available and where the attending physicians has to quickly manage all probable causes of the severe illness whilst waiting for definite laboratory confirmation. A study in Malawi in 2012 on adherence to national guidelines for managing severe malaria highlighted some of the practical issues facing prescribers in the management of severely ill patients and noted that initiation of appropriate treatment depends on availability of adequate resources [19].

In relation to testing for malaria itself, 71.8% of the patients in Ghana were tested using RDT or microscopy with a further 19.7% being tested with both RDT and microscopy. Just under 80% of the patients in Uganda were tested for presence of malaria parasites using either RDT or microscopy with less than 1% being tested using both RDT and microscopy. Nearly 20% of patients in Uganda and 10% of patients in Ghana were diagnosed clinically without parasitological confirmation by the use of RDT or microscopy. The WHO guidelines for the treatment of malaria as well as the practical handbook for Severe Malaria Management recommend the use of microscopy as part of the procedure for the diagnosis of severe malaria with a further recommendation that microscopy should be undertaken every 12 h during the 2–3 days of parenteral treatment to monitor response. None of the patients had 12-h microscopy to monitor treatment response.

Limitations of this study include the fact that the findings relate to prescriptions for patients in in-patient settings with no evidence that patients had been administered these medicines. Patients whose medications may be changed due to other considerations are still included in the analysis. Prescriptions that patients may already have but are unrelated to the current episode and admission may be missed. A drug utilisation study which examines availability of medicines in the treating facility as well as drug administration and its appropriateness in relation to age, weight and dose will be very helpful. In addition, the inclusion criteria for patients in this study was the prescription of parenteral anti-malarials (injectable AS, AR or Q) for treating presumed or confirmed severe malaria even though it may just represent overuse of injectable anti-malarials as found in previous studies. Further, this study did not look at provider related factors such as training on guidelines and health system related factors such as drug stock-outs which may influence the prescription patterns.

## Conclusion

Inj AS is the most commonly prescribed medicine in the management of severe malaria in Ghana and Uganda. However, adherence to the WHO guidelines which recommends the prescribing of injectable anti-malarial for at least 24 h followed by a full course of oral ACT is low.

## Abbreviations

ACT: artemisinin-based combination therapy
AQUAMAT: African Quinine Artesunate Malaria Trial
AS: artesunate
AR: artemether
Q: quinine
CRF: case report form
RDT: rapid diagnostic test
SEAQUAMAT: South East Asian Quinine Artesunate Malaria Trial
WHO: World Health Organization
